# MNS16A Tandem Repeats Minisatellite of Human Telomerase Gene and Cancer Risk: A Meta-Analysis

**DOI:** 10.1371/journal.pone.0073367

**Published:** 2013-08-22

**Authors:** Xiaoping Xia, Rui Rui, Sheng Quan, Rong Zhong, Li Zou, Jiao Lou, Xuzai Lu, Juntao Ke, Ti Zhang, Yu Zhang, Li Liu, Jie Yan, Xiaoping Miao

**Affiliations:** 1 Clinical Laboratory of Sir Run Run Shaw Hospital, Zhejiang University School of Medicine, Hangzhou, Zhejiang Province, China; 2 State Key Laboratory of Environment Health (Incubation), MOE (Ministry of Education) Key Laboratory of Environment & Health, Ministry of Environmental Protection Key Laboratory of Environment and Health (Wuhan), and Department of Epidemiology and Biostatistics, School of Public Health, Tongji Medical College, Huazhong University of Science and Technology, Wuhan, China; 3 Departments of Clinical Medicine, Zhejiang University City College, Hangzhou, Zhejiang Province, China; 4 Departments of Epidemiology and Biostatistics, School of Public Health, Guangdong Pharmaceutical University, Guangzhou, China; 5 Faculty of Basic Medicine, Zhejiang University School of Medicine, Hangzhou, Zhejiang Province, China; Sanjay Gandhi Medical Institute, India

## Abstract

**Background:**

Researchers have provided evidence that telomere dysfunction play an important role in cancer development. MNS16A is a polymorphic tandem repeats minisatellite of human telomerase (hTERT) gene that influences promoter activity of hTERT and thus implicates to relate with risk of several malignancies. However, results on association between MNS16A and cancer risk remain controversial. We therefore conduct a meta-analysis to derive a more precise estimation of association between MNS16A and cancer risk.

**Methods:**

A systematic literature search was conducted by searching PubMed, ISI Web of Knowledge, Human Genome and Epidemiology Network Navigator and Google Scholar digital database for publications on associations between MNS16A and cancer risk. Variants with statistically significant associations by meta-analysis were assessed using Venice criteria.

**Results:**

10 case-control articles enrolling 6101 cases and 10521 controls were brought into our meta-analysis. The relationships were strong epidemiological credibility in cerebral cancer and breast cancer population (*P* for heterogeneity > 0.1). The cumulative analysis in chronologic order suggested a clear tendency towards a significant association with additional study samples.

**Conclusions:**

The results provided a more accurate depiction of the role of MNS16A in cerebral cancer and breast cancer susceptibility. Additional larger studies were warranted to validate our findings.

## Introduction

Telomeres (a distinctive DNA-protein structure at the distal end of eukaryotic chromosomes) are crucial for genomic stability [1]–[5]. Somatic cells have a progressive shortening of telomeres after each cell division, however, telomeres reach a critical short length and lose capping function at the senescence stage in immortal tumor cells. Uncapped chromosomal ends will then trigger DNA-damage-like responses [6], [7]. The expressions of telomerase can prevent the loss of telomeres [8]–[10]. Human telomerase reverse transcriptase (hTERT) as the key constituent of telomerase, is highly expressed in essentially all immortal tumor cells, but is restricted in normal tissues, leading investigators to considerate hTERT as a critical role with cancer susceptibility [11]–[13]. MNS16A, a polymorphic tandem repeats minisatellite in downstream of hTERT gene, has been first reported to affect promoter activity in lung cancer cell lines [14]. The variants containing short tandem repeats (S allele) have stronger promoter activity than long repeats (L allele), indicating number of tandem repeats associated with lung cancer risk. Subsequently, several malignancies such as cerebral [15], [16], lung [17], [18], breast [19], [20], colorectal [21], nasopharyngeal [22], prostate cancer [23] and one meta-analysis [24] had investigated MNS16A in the etiology of cancer but with inconsistent results. Considering the important role of MNS16A in promoter activity of hTERT gene, we therefore conduct a meta-analysis on eligible articles to estimate association of MNS16A with cancer risk.

## Materials and Methods

### Search strategy, eligibility criteria and data extraction

All methodology was based on guidelines proposed by the Human Genome Epidemiology Network (HuGENet) [25] and the Preferred Reporting Items for Systematic Reviews and Meta-Analyses (PRISMA) [26] for systematic review of genetic association studies. A systematic review of original publications analyzing the association between MNS16A and cancer risk was performed by searching PUBMED, ISI Web of knowledge and Google Scholar database on and before February 2013, without language restriction. The strategy of keywords were: ("Neoplasm" [Mesh] OR "Carcinoma"[Mesh]) AND ("Telomerase"[Mesh] OR hTERT) AND MNS16A. Furthermore, we screened the Human Genome and Epidemiology Network Navigator as well as the references lists of key studies and reviews for additional publications [27]. We then performed the following criteria for literature selection: (a) original relevant case-control articles were included in this paper; (b) articles dealing with association between MNS16A and cancers in humans were available; (c) articles providing sufficient data to calculate ORs and 95% confidence intervals (CIs) were considered eligible. Information was extracted independently by two investi­gators (Rui and Zou) to ensure homogeneity of data collection and to rule out subjectivity effect in data gath­ering and entry. The following data should be noted: first author’s name, published year, location where the study was conducted, ethnicity, study period, mean age of case and control, source population, cancer type, sample size, variant counts in both cases and controls. For studies investigating more than one type of cancer, data were extracted separately as independent study [15], [16].

### Statistical analysis

#### Meta-analysis

For statistical analysis, number of tandem repeats was classified as either short (S) or long (L) alleles (LS classification system): S alleles, 213bp, 240bp, 243bp, 271bp, 272bp, 274bp; L alleles, 299bp, 302bp, 331bp, 333bp, 364bp, frequently applied in literature. On basis of classification, MNS16A genotypes were assigned to SS, LS or LL genotype groups. ORs and 95% CIs were recalculated and assessed in gene models based on MNS16A length comparisons (S allele versus L allele): a co dominant genetic model (SS versus LL; LS versus LL), a dominant genetic model (SS+LS versus LL) and a recessive model (SS versus LS + LL). To explore in depth of different lengths of MNS16A under S allele group, we classified the 271bp, 272bp and 274bp allele as middle alleles (M allele) and 213bp, 240bp and 243bp alleles still as S alleles (LMS classification system) described by Jin et al [18].

#### Sensitivity analyses and between-study heterogeneity

Between-study heterogeneity was assessed by the *χ2-*based Cochran’s *Q* statistic test and *I^2^* metric [28]. Heterogeneity was considered significant at *P*<0.1 for the *Q* statistic (to assess whether observed variance exceeds expected variance). And for the *I^2^* metric (*I^2^* = 100% × (*Q*-*df*)/*Q*), the following cut-off points were used: *I^2^* = 0–25%, no heterogeneity; *I^2^* = 25–50%, moderate heterogeneity; *I^2^* =  50–75%, large heterogeneity; *I^2^* = 75–100%, extreme heterogeneity. The significance of the combined ORs was determined using the *Z* test (*P*<0.05 was considered statistically significant). The DerSimonian and Laird random effect model [29] was used to calculate pooled ORs and 95% CIs according to their heterogeneity, otherwise, a fixed effects model (the Mantel-Haenszel method) was applied. Stratified analysis was performed for two ethnicity groups in order to investigate the hypothesis of ethnicity-specific genetic mechanisms in the development of MNS16A. Summary ORs and 95% CI were also calculated after stratification for cancer type. Additionally, sensitivity analysis was performed consecutively by omitting every article from the meta-analysis in turn to determine the influence of each study on the overall estimate [30]. Cumulative meta-analysis was performed through an assortment of all eligible cancer studies within the publication years. Finally, publication bias was evaluated by Begg’s test and Egger’s test to detect the small study effect [31]. All statistical analyses were performed with STATA software (version 10.1), and a 2-sided *P* value of less than 0.05 was considered significant, except for *Q* test for heterogeneity, for which a less than 0.1 level of statistical significance was applied.

#### Estimating the credibility of statistically significant associations

Each variant with statistically significant associations by meta-analysis were assessed on the basis of the Human Genome Epidemiology Network Venice criteria. Credibility was defined as “strong,” “moderate,” or “weak” based on grades A, B, or C in three categories: 1) amount of evidence; 2) replication; and 3) protection from bias. Amount of evidence was assessed by size of test allele among case and controls in meta-analysis (n_minor_): grade A, B, C requires n_minor_ > 1000, 100 ≤ n_minor_ ≤ 1000, n_minor_<100. Replication was graded by the heterogeneity statistic: grades A, B, and C were assigned for *I^2^* less than 25%, 25–50%, and greater than 50%, respectively. Assessment of protection from bias was graded as grade A if there was no observable bias, grade B if bias could be present, or grade C if bias was evidence ( the presence of a summary ORs less than 1.15 or loss of statistical significance after excluding the initial study) [32].

## Results

### Subjects characteristics

**Figure 1:**
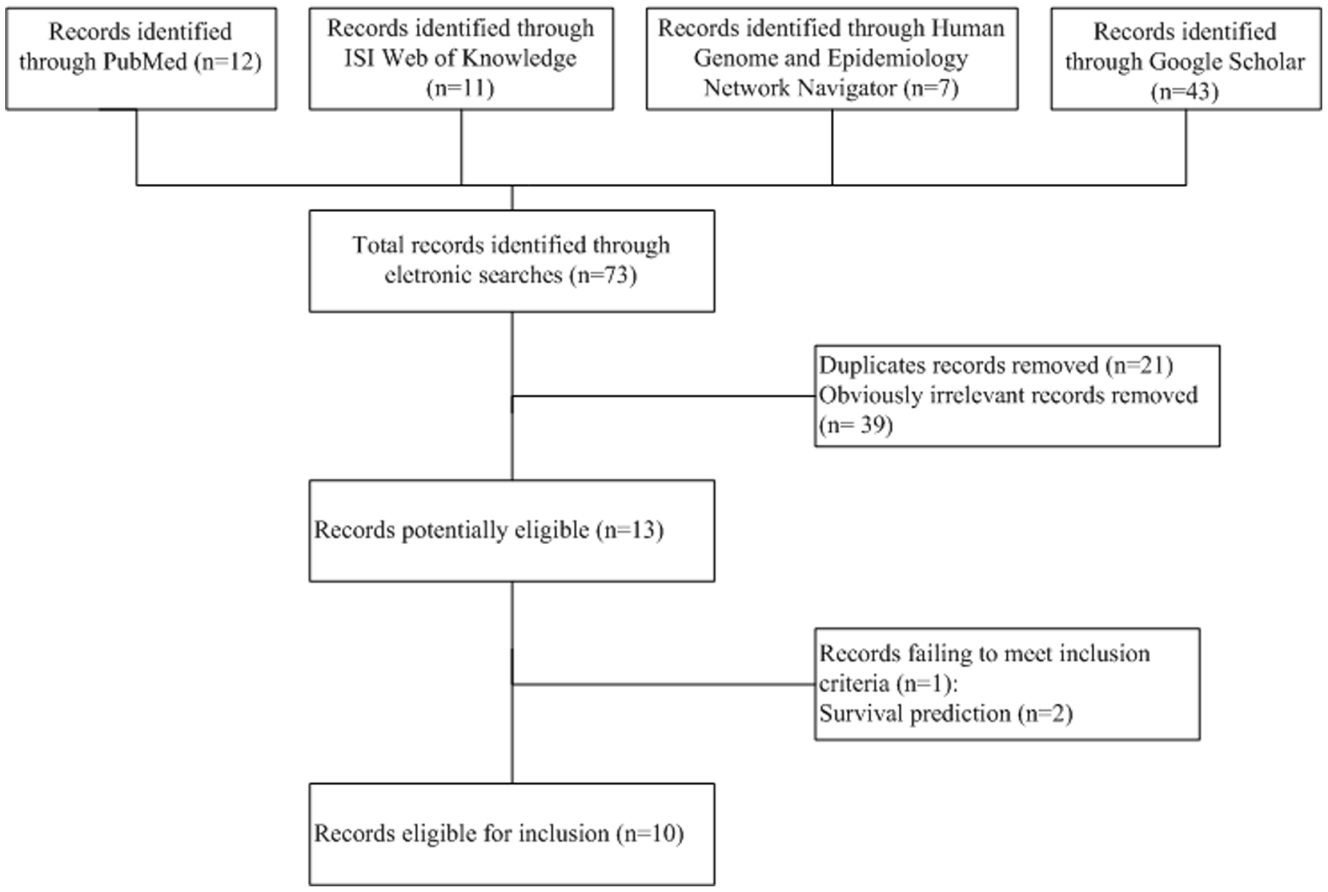
Flow chart of study selection.

After comprehensive searching of 71 articles, we identified 10 relevant publications including 6101 cases and 10521 controls from 13 studies to assess the association between MNS16A and cancer risk (Figure 1): 2 studies focused on glioblastoma [15], [16], 2 studies focused on glioma [15], [16], 3 studies focused on non-small cell lung cancer [14], [17], [18], 2 studies focused on breast cancer[19], [20] and each was one for meningioma [15], colorectal carcinoma [21], nasopharyngeal carcinoma [22] and prostate cancer [23] (Table 1). All studies were case-control studies, of which the most frequently investigated was brain cancer (6451 subjects; 38.81%). Among these, 9 studies were conducted in Caucasians (10400 subjects; 62.57%) and 4 in Asians (6222 subjects; 37.43%).

**Table 1:** Characteristics for case-control studies of MNS16A and risk of cancer included in a meta-analysis.

First author	Year	Study	Ethnicity	Mean	age	Source	Cancer	No. of	No. of	No. of	No. of
		location		case	control	population	type	case/	LL^a^	LS^b^	SS^c^
								control	case/	case/	case/
									control	control	control
Wang [14]	2003	USA	Caucasian	65.5	54.9	hospital	NSCLC	53/72	30/33	17/29	6/10
Carpentier[16]	2007	France	Caucasian	56.3	49.0	population	GBM	205/305	69/133	111/144	25/28
				46.25	49.0		Glioma	147/305	57/133	63/144	27/28
Wang [19]	2008	China	Asian	51.71	51.77	population	BC	1006/1095	860/984	141/107	5/4
Andersson[15]	2009	Europe	Caucasian	47	51	population	Glioma	648/1359	282/650	277/560	89/149
				52	51		Meningioma	473/1359	212/650	207/560	54/149
				NA	NA		GBM	291/1359	120/650	127/560	44/149
Jin [18]	2010	Korea	Asian	61.7	61.5	population	NSCLC	937/943	820/840	110/101	7/2
Hofer [21]	2011	Austria	Caucasian	66.8	61.3	population	CRC	88/1712	36/770	44/747	8/195
Zhang[22]	2011	China	Asian	NA	NA	population	NPC	798/1019	725/891	71/121	2/7
Chang[17]	2011	Taiwan	Asian	67.58	67.09	population	NSCLC	205/219	181/197	24/21	0/1
Zagouri[20]	2012	Greece	Caucasian	55.1	55.7	hospital	BC	113/124	50/63	36/29	27/32
Hofer [23]	2013	Austria	Caucasian	63.8	67.4	hospital	PC	1137/650	501/308	499/277	137/65

a,b,c The length of MNS16A were defined as L allele or S allele under LS classification system.

Abbreviation: NA, none anonymous; GBM, glioblastoma; BC, breast cancer; NSCLC, non-small cell lung cancer; CRC, colorectal cancer; NPC, nasopharyngeal cancer; PC, prostate cancer.

### Results of the meta-analysis

As shown in Table 2, all studies were pooled into a meta-analysis, and the increased association between MNS16A and cancer risk were found for all genotypic models. Random-effect model pooling analyses yielded overall ORs of 1.15 (95% CI = 1.03–1.28; *P* for heterogeneity = 0.102, *I^2^* = 35.0%) for LS genotype versus LL genotype, and 1.17 (95%CI = 1.05–1.31; *P* for heterogeneity = 0.064, *I^2^* = 40.5%) for dominant model. In fixed-effects model, overall ORs were 1.32 (95%CI = 1.14–1.53; *P* for heterogeneity = 0.337, *I^2^* = 10.8%) for SS genotype versus LL genotype, and 1.23 (95% CI = 1.07–1.41; *P* for heterogeneity = 0.307, *I^2^* = 13.7%) for recessive model.

**Table 2:** Pooled ORs with 95% CIs for the association between MNS16A and cancer risk in meta-analysis.

Category	Genetic model	ORs (95% CI)	*P* a	*P* for	*I^2^*
				Heterogeneity	
LS classification	S vs. L	1.13 (1.03–1.25)	0.013	0.012	53.3%
(No. of study = 13)	LS vs. LL	1.15 (1.03–1.28)	0.015	0.102	35.0%
	SS vs. LL	1.32 (1.14–1.53)	0.000	0.337	10.8%
	Dominant	1.17 (1.05–1.31)	0.006	0.064	40.5%
	Recessive	1.23 (1.07–1.41)	0.003	0.307	13.7%
LMS classification b	S vs. L	1.21 (1.04–1.41)	0.015	0.047	50.8%
(No. of study = 8)	M vs. L	1.04 (0.75–1.42)	0.830	0.041	52.1%
	LM+MM vs. LL	1.04 (0.73–1.50)	0.823	0.003	54.8%
	LS+MS+SS vs. LL	1.75 (1.02–1.73)	0.041	0.000	93.0%
	LS+MS+SS vs. LL+LM+MM	1.03 (0.73–1.45)	0.862	0.000	79.3%

a *P* value was calculated by the *Z* test.

b The length of MNS16A was defined as L, M or S allele under LMS classification system.

Subsequently we categorized the data in LMS classification described by Jin et al. to explore in depth the effect of MNS16A S allele (the short allele) and M allele (the middle allele) with cancer risk. As shown in Table 2, 8 studies were classified during LMS classification system. All genetic models revealed that S allele presented a great cancer risk than M allele and 95%CIs were nearby statistically significant.

### Stratified analysis

**Figure 2:**
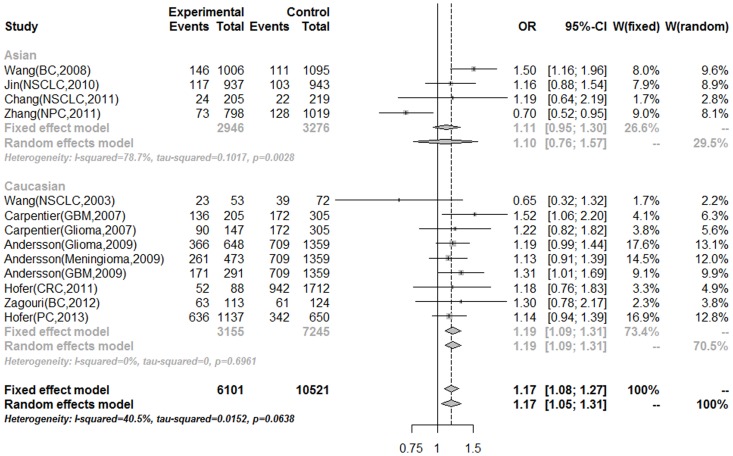
Forest plot of MNS16A association with cancer risk under dominant model stratified by ethnicity.

Stratified analysis was performed for two ethnicity groups in order to investigate the hypothesis of Asian and Caucasian genetic mechanisms in the development of MNS16A. (Table 3). No evidence of heterogeneity was revealed in Caucasian population (*P* for heterogeneity > 0.1), and all genetic models presented a significantly increased cancer risk, with ORs of 1.16 (95%CI = 1.05–1.28), 1.33 (95%CI = 1.15–1.54), 1.19 (95%CI = 1.09–1.31), and 1.23 (95%CI = 1.07–1.42) for LS versus LL genotype, SS versus LL genotype, dominant model, and recessive model, respectively. However, all genetic models presented no statistical differences of cancer risk among Asian population (Figure 2).

**Table 3:** Pooled ORs with 95% CIs for the association between MNS16A and cancer risk by stratified analysis.

	Category	Genetic model	ORs (95%CI)	*P* a	*P* for heterogeneity	*I^2^*
Ethnicity	Caucasian	S vs. L	1.16 (1.06–1.26)	0.001	0.235	23.4%
	(No. of study = 9)	LS vs. LL	1.16 (1.05–1.28)	0.003	0.689	0.00%
		SS vs. LL	1.33 (1.15–1.54)	0.000	0.383	6.20%
		Dominant	1.19 (1.09–1.31)	0.000	0.696	0.00%
		Recessive	1.23 (1.07–1.42)	0.003	0.322	13.5%
	Asian	S vs. L	1.08 (0.76–1.55)	0.658	0.002	80.0%
	(No. of study = 4)	LS vs. LL	1.10 (0.78–1.56)	0.591	0.005	76.3%
		SS vs. LL	1.13 (0.54–2.35)	0.747	0.188	37.3%
		Dominant	1.10 (0.76–1.57)	0.621	0.003	78.7%
		Recessive	1.12 (0.53–2.33)	0.768	0.204	34.8%
Cancer type	Cerebral Cancer	S vs. L	1.19 (1.10–1.30)	0.000	0.503	0.00%
	(No. of study = 5)	LS vs. LL	1.17 (1.04–1.32)	0.008	0.708	0.00%
		SS vs. LL	1.42 (1.19–1.70)	0.000	0.303	17.6%
		Dominant	1.22 (1.09–1.37)	0.000	0.686	0.00%
		Recessive	1.32 (1.11–1.56)	0.001	0.248	26.0%
	Lung Cancer	S vs. L	0.96 (0.62–1.49)	0.842	0.066	63.3%
	(No. of study = 3)	LS vs. LL	1.07 (0.84–1.37)	0.571	0.379	0.00%
		SS vs. LL	1.14 (0.51–2.57)	0.744	0.180	41.7%
		Dominant	1.07 (0.81–1.42)	0.627	0.315	13.4%
		Recessive	1.23 (0.56–2.73)	0.609	0.229	32.2%
	Breast Cancer	S vs. L	1.31 (1.00–1.72)	0.046	0.214	35.2%
	(No. of study = 2)	LS vs. LL	1.52 (1.19–1.94)	0.001	0.914	0.00%
		SS vs. LL	1.12 (0.64–1.99)	0.687	0.691	0.00%
		Dominant	1.46 (1.16–1.84)	0.002	0.620	0.00%
		Recessive	0.97 (0.57–1.66)	0.904	0.576	0.00%

a *P* value was calculated by the *Z* test.

**Figure 3:**
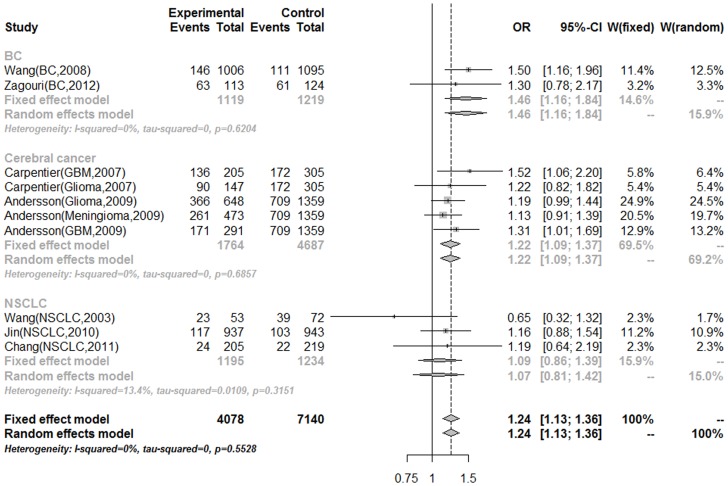
Forest plot of MNS16A association with cancer risk under dominant model stratified by cancer type.

Then, we assessed the source of heterogeneity by cancer type (Table 3). On the basis of five cerebral cancer studies, there was no heterogeneity for all genetic models *(P* for heterogeneity > 0.1). Patients with MNS16A-S allele had a significant statistically association with cerebral cancer risk: with ORs of 1.42 (95%CI = 1.19–1.70), 1.22 (95%CI = 1.09–1.37), 1.32 (95%CI = 1.11–1.56) for SS versus LL genotype, dominant and recessive model (*P* for heterogeneity > 0.1). For breast cancer, patients carried with LS genotype had higher risk than SS genotype, which ORs and 95%CI were 1.52 (1.19–1.94) and 1.46 (1.16–1.84) for LS versus LL genotype and dominant models. However, no statistically significant associations were observed with lung cancer patients (Figure 3).

### Cumulative meta-analysis

**Figure 4:**
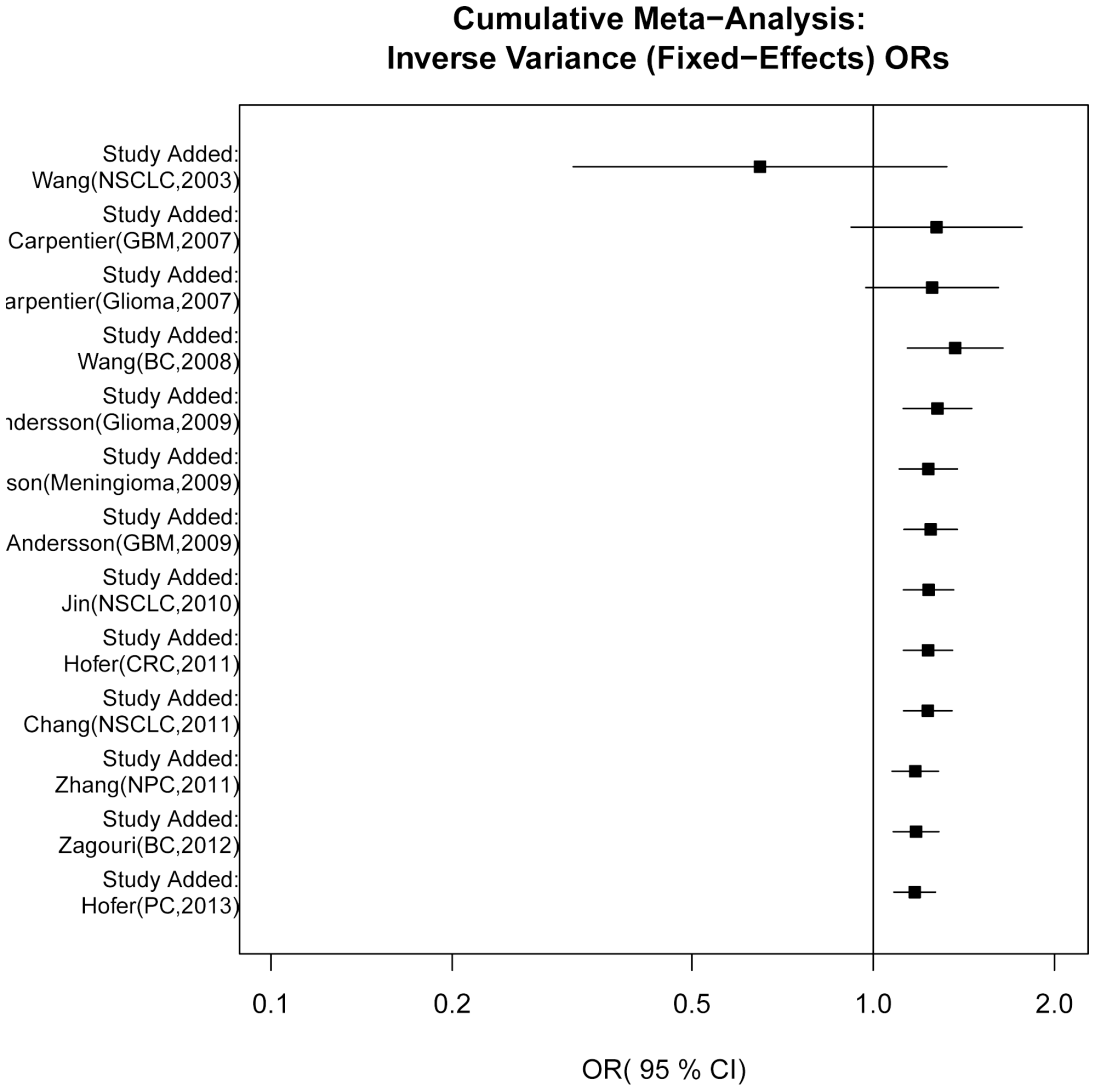
Cumulative meta-analysis of association MNS16A with risk of cancer under dominant model.

Cumulative meta-analyses of MNS16A were conducted via an assortment of studies in chronologic order. Figure 4 shows the results from the cumulative meta-analyses in fixed-effects model. The effect of MNS16A tended to show a significant association over time in all genetic models. Moreover, the 95% CIs became increasingly narrow with increasing data, suggesting that the precision of the estimates was progressively boosted by continually adding more studies.

### Sensitivity analysis

Since moderate heterogeneity was observed under the genotypic model of LS versus LL and dominant models, we conducted a sensitivity meta-analysis to assess effects of each study on the combined ORs and 95% CIs. A random-effect model was employed since heterogeneity was indicated. Sensitivity analysis indicated the independent study contributing the most heterogeneity was conducted by Zhang et al. The heterogeneity was completely reduced by exclusion of that study: under the genotypic model of LS versus LL, ORs = 1.15 (95%CI = 1.03–1.28, *P* for heterogeneity  = 0.102, *I^2^* = 35.0%) and ORs = 1.20 (95%CI = 1.10–1.31, *P* for heterogeneity  = 0.656, *I^2^* = 0.00%) before and after removal, respectively. Omission of studies by Andersson et al. changed the pooled ORs fractionally (Table 4).

**Table 4:** Pooled ORs with 95% CIs for the association between MNS16A and cancer risk by omitting each article in sensitivity analysis.

First author	Year	Genetic model	ORs (95%CI)	*P* a	*P* for	*I^2^*
					heterogeneity	
Wang [14]	2003	LS vs. LL	1.16 (1.07–1.27)	0.000	0.136	31.8%
		Dominant	1.19 (1.07–1.33)	0.001	0.097	36.7%
Carpentier [16]	2007	LS vs. LL	1.14 (1.01–1.29)	0.028	0.091	38.8%
		Dominant	1.15 (1.02–1.30)	0.023	0.053	44.9%
Wang [19]	2008	LS vs. LL	1.12 (1.03–1.23)	0.009	0.224	22.3%
		Dominant	1.15 (1.06–1.25)	0.001	0.122	33.5%
Andersson [15]	2009	LS vs. LL	1.15 (0.97–1.36)	0.103	0.034	50.3%
		Dominant	1.15 (0.98–1.37)	0.187	0.023	53.4%
Jin [18]	2010	LS vs. LL	1.16 (1.03–1.31)	0.017	0.076	39.8%
		Dominant	1.18 (1.04–1.33)	0.009	0.044	45.3%
Hofer [21]	2011	LS vs. LL	1.15 (1.02–1.29)	0.019	0.077	39.5%
		Dominant	1.18 (1.04–1.32)	0.007	0.044	45.3%
Zhang [22]	2011	LS vs. LL	1.20 (1.10–1.31)	0.000	0.656	0.00%
		Dominant	1.23 (1.13–1.33)	0.000	0.719	0.00%
Chang [17]	2011	LS vs. LL	1.15 (1.03–1.29)	0.016	0.075	39.8%
		Dominant	1.18 (1.05–1.32)	0.006	0.044	45.3%
Zagouri [20]	2012	LS vs. LL	1.15 (1.02–1.28)	0.018	0.097	36.7%
		Dominant	1.17 (1.04–1.31)	0.008	0.046	45.0%
Hofer [23]	2013	LS vs. LL	1.15 (1.02–1.31)	0.027	0.074	40.0%
		Dominant	1.17 (1.03–1.33)	0.015	0.044	45.3%

a *P* value was calculated by the *Z* test.

### Publication bias

As reflected by either visualization of funnel plot or Egger’s and Begg’s test, there was no indication of publication bias in the genotypic models of LS versus LL, SS versus LL, dominant, and recessive model (*P* = 0.482, *P* = 0.537, *P* = 0.551, and *P* = 0.745, respectively), indicating the results were statistically robust.

### Grading of associations

Based on the previously proposed guidelines and applying the Venice criteria, the amount of evidence was categorized as A, since its n_minor_ is above 1,000 (n_minor_ = 2558); replication was assigned to category B, because the amount of between-study heterogeneity (*I^2^* =  40.5%); and protection from bias was graded as category B, due to the presence of summary ORs less than 1.15, which can easily be dissipated even by relatively small biases in a meta-analysis of published data. The overall assessment of association between MNS16A and cancer risk were moderate cumulative evidence (ABB). After stratification by ethnicity, the meta-analysis consistently showed a significant association cancer risk in Caucasian population and were assigned an overall strong epidemiological credibility (AAA). Asian population lacked of statistically significant findings and was placed to weak evidence. In addition, strong epidemiological credibility (AAA) was also observed for association between MNS16A with cerebral cancer and breast cancer (Table 5).

**Table 5:** Assessment of cumulative evidence for the association of MNS16A dominant model and risk of cancer.

	No. of	No. of	ORs	*P* for	*P* for	*I^2^*	Level of evidence
	Case	Control	95% CI	Z test	Heterogeneity		
Overall	6101	10521	1.17 (1.05–1.31)	0.006	0.064	40.5%	ABB/Moderate
Caucasian	3155	7245	1.19 (1.09–1.31)	0.000	0.696	0.00%	AAA/Strong
Asian	2946	3276	1.10 (0.76–1.57)	0.621	0.003	78.7%	Weak
Cerebral Cancer	1263	2974	1.22 (1.09–1.37)	0.000	0.686	0.00%	AAA/Strong
Lung Cancer	177	157	1.07 (0.81–1.42)	0.627	0.229	32.2%	Weak
Breast Cancer	1119	1219	1.46 (1.16–1.84)	0.002	0.620	0.00%	AAA/Strong

Abbreviation: NSCLC, non-small cell lung cancer; A, B, and C represent the Venice criteria grades for amount of evidence, replication of association and protection from bias, which ultimately define the level of cumulative evidence (strong, moderate, weak).

### Comparison with previously published meta-analyses

In a meta-analysis that regarding all hTERT locus polymorphisms with cancer susceptibility, Simone et al. investigated that MNS16A S allele was statistically associated with increased risk of central nervous system tumors (CNS). In comparison, our meta-analysis added more publications to consider association of MNS16A with all available type of cancer; analyzed data in different MNS16A classification system (LS and LMS classification system); stratified ethnicity and cancer types for further research.

## Discussion

A number of well designed genome wide association studies (GWAS) had implicated variants at hTERT locus to be significantly associated with almost all malignant tumors [33]. MNS16A, a 23 bp (or 26 bp) tandem repeat sequence (TCCTCTTAT (cat) CTCCCAGTCTC) in putative promoter region of the antisense RNA transcript, was first reported to increase expression of hTERT mRNA in lung cancer tissues. In the current study, we conducted a meta-analysis of 10 previously published articles comprising 6101 cases and 10521 controls concerning association of MNS16A with cancer risk. Although all genetic models of MNS16A showed a moderate association with cancer risk, the effect could very well be driven by the effect on cerebral cancer. Thereupon, we stratified cancer types and found cerebral cancer and breast cancer patients showed strong cumulative evidence for associations, but lung cancer was not. Apart from this, ethnicity was also stratified in this work. Caucasian population presented a significantly increased relationship with cancer risk, whereas Asians not. The variance of effect between Caucasians and Asians might be contribute to that approximate 70% Caucasians were cerebral cancer, while similarly the absence of effect in Asians might well be due to the fact that only non-cerebral cancer were carried in this population. In addition, there was almost no obviously heterogeneity by stratified for cancer type, which suggested differential effects of MNS16A in diverse kinds of cancer. However, functional importance of the antisense transcript activity and exact molecular mechanisms of MNS16A with different cancer types were still unclear.

In this work, we analyzed data in different classification system: LS and LMS (described by Hofer et al. [21]) classification system for further excavation. The results figured that S allele had higher relationship than M allele with MNS16A. The cause might due to length of MNS16A: M allele contains three 26 bp repeats; whereas S alleles contain two 26 bp repeats. Hence we could see that, 26 bp sequence may influence as a repressor for promoter of antisense TERT mRNA [18]. It is more reasonable to analysis MNS16A S allele and M allele separately in future research to find accurate genotype with cancer risk.

Through sensitivity analysis, omission of one article by Zhang et al. eliminated heterogeneity of LS versus LL genotype and dominant models (P for heterogeneity > 0.1). The reason might due to lower frequencies of S allele in Asians. Additionally, omission article by Carpentier, the ORs were still presented increased risk, and 95%CI were nearby statistically significant (OR = 1.15, 95%CI =  1.03–1.28; OR = 1.14, 95%CI = 1.01–1.29, before and after removal), which not meaningfully changed the pooled ORs, as well as the article by Andersson.

Some limitations needed serious consideration. First, our result was based on unadjusted estimates. Individual data were not available for an adjusted estimate by age and sex, which might potentially lead to false positive results. Another limitation was lacking original data to limit our further evaluation of gene-environment interaction such as smoking, alcohol use and other clinical characteristics. Finally, lacking of sufficient original studies limited our further evaluation of colorectal cancer, breast cancer and nasopharyngeal carcinoma risk with MNS16A.

## Conclusion

This work verified the important role of MNS16A minisatellites in cerebral and breast cancer predisposition. Additional larger studies were warranted to validate our findings.

## Supporting Information

Checklist S1 (DOC)

## References

[1] Greider CW (1991) Chromosome first aid. Cell 67: 645–647. 1934066 10.1016/0092-8674(91)90058-7

[2] Murnane JP (2006) Telomeres and chromosome instability. DNA Repair (Amst) 5: 1082–1092. 16784900 10.1016/j.dnarep.2006.05.030

[3] Ju Z, Rudolph KL (2006) Telomeres and telomerase in cancer stem cells. Eur J Cancer 42: 1197–1203. 16644207 10.1016/j.ejca.2006.01.040

[4] Cong YS, Wright WE, Shay JW (2002) Human telomerase and its regulation. Microbiol Mol Biol Rev 66: : 407–425, table of contents. 10.1128/MMBR.66.3.407-425.2002 PMC120798 12208997

[5] Blackburn EH (2001) Switching and signaling at the telomere. Cell 106: 661–673. 11572773 10.1016/s0092-8674(01)00492-5

[6] Satyanarayana A, Manns MP, Rudolph KL (2004) Telomeres and telomerase: a dual role in hepatocarcinogenesis. Hepatology 40: 276–283. 15368430 10.1002/hep.20308

[7] Vaziri H, Benchimol S (1996) From telomere loss to p53 induction and activation of a DNA-damage pathway at senescence: the telomere loss/DNA damage model of cell aging. Exp Gerontol 31: 295–301. 8706799 10.1016/0531-5565(95)02025-x

[8] Kim NW, Piatyszek MA, Prowse KR, Harley CB, West MD, et al (1994) Specific association of human telomerase activity with immortal cells and cancer. Science 266: 2011–2015. 7605428 10.1126/science.7605428

[9] Broccoli D, Young JW, de Lange T (1995) Telomerase activity in normal and malignant hematopoietic cells. Proc Natl Acad Sci U S A 92: 9082–9086. 7568077 10.1073/pnas.92.20.9082 PMC40928

[10] Lantuejoul S, Salon C, Soria JC, Brambilla E (2007) Telomerase expression in lung preneoplasia and neoplasia. Int J Cancer 120: 1835–1841. 17311257 10.1002/ijc.22473

[11] Wick M, Zubov D, Hagen G (1999) Genomic organization and promoter characterization of the gene encoding the human telomerase reverse transcriptase (hTERT). Gene 232: 97–106. 10333526 10.1016/s0378-1119(99)00108-0

[12] Feng J, Funk WD, Wang SS, Weinrich SL, Avilion AA, et al (1995) The RNA component of human telomerase. Science 269: 1236–1241. 7544491 10.1126/science.7544491

[13] Stanta G, Bonin S, Niccolini B, Raccanelli A, Baralle F (1999) Catalytic subunit of telomerase expression is related to RNA component expression. FEBS Lett 460: 285–288. 10544251 10.1016/s0014-5793(99)01357-5

[14] Wang L, Soria JC, Chang YS, Lee HY, Wei Q, et al (2003) Association of a functional tandem repeats in the downstream of human telomerase gene and lung cancer. Oncogene 22: 7123–7129. 14562040 10.1038/sj.onc.1206852

[15] Andersson U, Osterman P, Sjostrom S, Johansen C, Henriksson R, et al (2009) MNS16A minisatellite genotypes in relation to risk of glioma and meningioma and to glioblastoma outcome. Int J Cancer 125: 968–972. 19405125 10.1002/ijc.24363

[16] Carpentier C, Lejeune J, Gros F, Everhard S, Marie Y, et al (2007) Association of telomerase gene hTERT polymorphism and malignant gliomas. J Neurooncol 84: 249–253. 17410334 10.1007/s11060-007-9378-3

[17] Chang CC, Yu MC, Bai KJ, Chang JH, Lee CN, et al (2011) The Analysis Between Functional Human Telomerase Reverse Transcriptase MNS16A Polymorphisms and the Risk of Developing Non-Small Cell Lung Cancer in the Taiwanese Population. Journal of Experimental and Clinica l Medicine 3: 3.

[18] Jin G, Yoo SS, Cho S, Jeon HS, Lee WK, et al (2011) Dual roles of a variable number of tandem repeat polymorphism in the TERT gene in lung cancer. Cancer Sci 102: 144–149. 21083788 10.1111/j.1349-7006.2010.01782.x PMC11159479

[19] Wang Y, Hu Z, Liang J, Wang Z, Tang J, et al (2008) A tandem repeat of human telomerase reverse transcriptase (hTERT) and risk of breast cancer development and metastasis in Chinese women. Carcinogenesis 29: 1197–1201. 18413362 10.1093/carcin/bgn099

[20] Zagouri F, Sergentanis TN, Gazouli M, Tsigginou A, Dimitrakakis C, et al (2012) HTERT MNS16A polymorphism in breast cancer: a case-control study. Mol Biol Rep 39: 10859–10863. 23065203 10.1007/s11033-012-1982-4

[21] Hofer P, Baierl A, Feik E, Fuhrlinger G, Leeb G, et al (2011) MNS16A tandem repeats minisatellite of human telomerase gene: a risk factor for colorectal cancer. Carcinogenesis 32: 866–871. 21422235 10.1093/carcin/bgr053 PMC3314280

[22] Zhang Y, Zhang H, Zhai Y, Wang Z, Ma F, et al (2011) A functional tandem-repeats polymorphism in the downstream of TERT is associated with the risk of nasopharyngeal carcinoma in Chinese population. BMC Med 9: 106. 21929825 10.1186/1741-7015-9-106 PMC3191471

[23] Hofer P, Zerelles J, Baierl A, Madersbacher S, Schatzl G, et al.. (2013) MNS16A tandem repeats minisatellite of human telomerase gene and prostate cancer susceptibility. Mutagenesis. 10.1093/mutage/get003 23423318

[24] Mocellin S, Verdi D, Pooley KA, Landi MT, Egan KM, et al (2012) Telomerase reverse transcriptase locus polymorphisms and cancer risk: a field synopsis and meta-analysis. J Natl Cancer Inst 104: 840–854. 22523397 10.1093/jnci/djs222 PMC3611810

[25] Sagoo GS, Little J, Higgins JP (2009) Systematic reviews of genetic association studies. Human Genome Epidemiology Network. PLoS Med 6: e28. 19260758 10.1371/journal.pmed.1000028 PMC2650724

[26] Swartz MK (2011) The PRISMA statement: a guideline for systematic reviews and meta-analyses. J Pediatr Health Care 25: 1–2. 21147401 10.1016/j.pedhc.2010.09.006

[27] Yu W, Gwinn M, Clyne M, Yesupriya A, Khoury MJ (2008) A navigator for human genome epidemiology. Nat Genet 40: 124–125. 18227866 10.1038/ng0208-124

[28] Higgins JP, Thompson SG (2002) Quantifying heterogeneity in a meta-analysis. Stat Med 21: 1539–1558. 12111919 10.1002/sim.1186

[29] DerSimonian R, Laird N (1986) Meta-analysis in clinical trials. Control Clin Trials 7: 177–188. 3802833 10.1016/0197-2456(86)90046-2

[30] Tobias A (1999) Assessing the influence of a single study in meta-analysis. Stata Technical Bulletin 8: 7.

[31] Egger M, Davey Smith G, Schneider M, Minder C (1997) Bias in meta-analysis detected by a simple, graphical test. BMJ 315: 629–634. 9310563 10.1136/bmj.315.7109.629 PMC2127453

[32] Langevin SM, Ioannidis JP, Vineis P, Taioli E (2010) Assessment of cumulative evidence for the association between glutathione S-transferase polymorphisms and lung cancer: application of the Venice interim guidelines. Pharmacogenet Genomics 20: 586–597. 20729793 10.1097/FPC.0b013e32833c3892 PMC2940992

[33] Baird DM (2010) Variation at the TERT locus and predisposition for cancer. Expert Rev Mol Med 12: e16. 20478107 10.1017/S146239941000147X

